# Chronic fatigue syndrome and fibromyalgia in diagnosed sleep disorders: a further test of the ‘unitary’ hypothesis

**DOI:** 10.1186/s12883-015-0308-2

**Published:** 2015-04-12

**Authors:** Slobodanka Pejovic, Benjamin H Natelson, Maria Basta, Julio Fernandez-Mendoza, Fauzia Mahr, Alexandros N Vgontzas

**Affiliations:** Department of Psychiatry, Sleep Research and Treatment Center, Penn State College of Medicine, Hershey, PA 17033 USA; Department of Neurology, Pain and Fatigue Study Center, Mount Sinai Beth Israel, Icahn School of Medicine at Mount Sinai, New York, NY 10003 USA; Department of Psychiatry, School of Medicine, University of Crete, Iraklion, Greece

**Keywords:** Fibromyalgia, Chronic fatigue syndrome, Obstructive sleep apnea, Insomnia, Sleepiness, Depression

## Abstract

**Background:**

Since chronic fatigue syndrome (CFS) and fibromyalgia (FM) often co-exist, some believe they reflect the same process, somatization. Against that hypothesis are data suggesting FM but not CFS was common in patients with sleep-disordered breathing (SDB). The presence of discrete case definitions for CFS and FM allowed us to explore rates of CFS alone, CFS with FM, and FM alone in SDB patients compared to those with sleep complaints that fulfilled criteria for insomnia.

**Methods:**

Participants were 175 sequential patients with sleep-related symptoms (122 had SDB and 21 had insomnia) and 39 healthy controls. Diagnoses were made by questionnaires, tender point count, and rule out labs; sleepiness was assessed with Epworth Sleepiness Scale and mood with Beck Depression Inventory.

**Results:**

Rates of CFS, FM or CFS + FM were high: 13% in SDB and 48% in insomnia. CFS occurred frequently in SDB and insomnia, but FM occurred frequently only in insomnia. SDB patients with CFS and/or FM had higher daytime sleepiness than those without these disorders.

**Conclusion:**

CFS patients should complete Epworth scales, and sleep evaluation should be considered for those with scores ≥ 16 before receiving the diagnosis of CFS; the coexistence of depressed mood in these patients suggests some may be helped by treatment of their depression. That FM was underrepresented in SDB suggests FM and CFS may have different underlying pathophysiological causes.

## Background

Chronic fatigue syndrome (CFS) and fibromyalgia (FM) are symptom-based illnesses, diagnosed using sets of clinical criteria agreed upon by experts [1,2]. However the core symptoms of pain, fatigue, sleep problems and cognitive difficulties exist across both syndromes and lead to significant co-morbidity between them. The fact that these two syndromes co-exist so often has led some to question whether they are, in fact, distinct diagnostic entities. Wessely et al. [3], for example, have suggested that the “similarities between them outweigh the differences” and Barsky and Borus [4], noting similarities between CFS and FM, suggested an explanatory model based on the concept of symptom amplification – that is, a common psychological tendency to somatize or misconstrue the significance of normal physical sensation. We have termed this the “unitary” hypothesis. If that hypothesis were true, discrete case definitions corresponding to distinct illness syndromes would be unnecessary. However, there is substantial evidence against this interpretation showing that CFS can differ from FM [5] – thus providing evidence against the unitary hypothesis. Finding these differences argues for the two illnesses being different – with different underlying pathophysiology.

The underlying causes of disturbed and/or unrefreshing sleep, common complaints in both CFS and FM, remain to be more fully considered – specifically as to whether rates of sleep pathology differ between CFS and FM. While untreated sleep apnea and narcolepsy were considered exclusions for the diagnosis of CFS, formal sleep evaluation including polysomnography (PSG) was not recommended in evaluating a patient with severe fatigue [1]. That decision seemed appropriate because, in contrast to early studies that suggested a high rate of sleep disorders in CFS [6-8], later studies including our own found no difference in rates of sleep disorders between patients with CFS and healthy controls [9-11]. In contrast, except for our own work [11] and one other early study [12], a number of other studies suggests that FM is often accompanied by sleep-disordered breathing (SDB) [13-16].

Existing case definitions allow for the diagnosis of either disorder occurring alone or CFS and FM occurring together. Because prior studies of symptom-based illness usually did not differentiate between CFS or FM [5], we thought that research on the relation among CFS alone, FM alone, CFS with FM and sleep diagnosis might further understanding about the pathophysiological underpinnings of these syndromes. To do this, we evaluated a consecutive cohort of patients with sleep complaints for these diagnoses; the patients then underwent diagnostic PSG. Based on the literature, we expected to find increased rates of FM but not of CFS in patients with SDB. Our plan was to compare these data to those in patients with insomnia, i.e., sleep complaints with PSG-based evidence of increased sleep latency, prolonged periods of wakefulness following sleep onset, or early morning awakening. Since disturbed sleep is a complaint common to both CFS and FM, we expected to find high rates of both these illnesses in these patients. Finally, we also evaluated patients for irritable bowel syndrome (IBS), because it is known to co-exist with FM and CFS [17] and thus could be an independent risk factor for sleep pathology.

## Methods

### Subjects

Participants were 175 patients and 39 healthy controls, aged ≥18 and body mass index (BMI) ≤40. Patients were a consecutive series of people with sleep-related symptoms (breath cessations, snoring, excessive daytime sleepiness, fatigue, difficulty initiating or maintaining sleep or early morning awakening) who underwent diagnostic polysomnography followed by evaluation by a sleep specialist between June 2006 and June 2007 at the Sleep Research and Treatment Center and were found to have a primary sleep disorder. A wide variety of practitioners in central Pennsylvania, i.e., primary care as well as specialty physicians’ offices, referred these patients in order to rule out primary sleep disorder; none of the patients was referred by the Pain & Fatigue Study Center in New Jersey. Controls were Dauphin county residents, taking no medications, in good health with neither sleep-related complaints nor wide spread pain. All subjects signed their informed consent to participate in this study which was approved by Penn State College of Medicine’s Institutional Review Board.

### Sequence of procedures

#### Assessment of fatigue and pain, sleepiness, and depression

On entry to the Sleep Research and Treatment Center, one of the medical doctors involved in this study (SP, MB, and FM) did a medical evaluation on each patient, including medical and sleep history, a physical examination including a tender point examination, and clinical tests (including complete cell blood count; liver and renal profiles; glucose; thyroid indices and an electrocardiogram). Patients were then asked to complete a questionnaire assessing medical and psychiatric symptoms developed and used extensively by one of the authors, BHN, to identify patients with CFS, fibromyalgia, and irritable bowel syndrome (http://www.painandfatigue.com/).

Immediately thereafter, daytime sleepiness and mood were assessed by Epworth Sleepiness Scale (ESS) and Beck Depression Inventory (BDI), respectively [18,19]. BDI results did not change when the items related to sleep disturbance and fatigue were eliminated from the total BDI score.

#### Diagnosis of FM and Chronic Fatigue Syndrome (CFS)

The diagnosis of FM was made using the 1990 case definition [2]: existence of chronic widespread pain plus tenderness on palpation in at least 11 of 18 tender point sites. Pain was considered chronic and widespread if patients indicated on the questionnaire that it existed over the past 3 months in: (1) shoulders, arms or hands on both left and right side of the body, (2) legs or feet on left and right sides, and (3) chest, neck or back on both left and right sides as reported in the intake questionnaire. A tender point was counted if the patient reported severity of pain during palpation with 4 kg of force to be ≥ 2 on a scale from 0 to 10, with 0 meaning no pain and 10 meaning worst pain possible [20].

Patients were diagnosed with chronic fatigue syndrome if they fulfilled the 1994 case definition [1]. Specifically, patients responded to a five point visual analog scale in the intake questionnaire to rate the degree fatigue had reduced their activity; to be included, patients had to indicate that their fatigue had had a substantial, severe, or very severe effect (ascertained by scores of ≥3 on a 0 to 5 scale where 3 was substantial, 4 severe and 5 very severe) on their level of activity on the job, in school or class, socially, or in their personal life. Next, patients were asked about the duration they had suffered from the following symptoms: sore throat, tender lymph glands, headache, myalgia, arthralgia, unrefreshing sleep, cognitive difficulties and the complaint that even minimal exertion produced a dramatic worsening of their entire symptom complex; to be included, patients had to report that at least 4 of these symptoms had been a problem for at least 6 months with severity in the month prior to intake rated as ≥3 on 0 to 5 visual analog scales where 0 was none, 1 mild, 2 moderate, 3 substantial, 4 severe, and 5 very severe. Finally, to receive the diagnosis of CFS, patients had to have normal results on laboratory tests. The existence of independent diagnostic criteria for CFS and for FM allowed the identification of patients fulfilling diagnostic criteria for either or both disorders.

#### Diagnosis of Irritable Bowel Syndrome (IBS)

Diagnosis of IBS required patients to fulfill ROME II criteria – i.e., at least 12 weeks, not necessarily consecutive, in the preceding year of abdominal discomfort or pain that had two of three features: relieved with defecation; onset associated with change in frequency of daily bowel movements from normal to >3/day or to <3/week; onset associated with a change in form from normal to lumpy/hard or to loose/watery in the intake questionnaire [21].

#### Sleep laboratory recordings

Immediately after these procedures, subjects were evaluated for one night for 8 h (2300–0700) in sound attenuated, light and temperature controlled rooms following standard polysomnographic procedures with continuous multi-channel monitoring of electroencephalogram (3 channels), electro-oculogram (3 channels), and electromyogram (1 channel). Sleep records were subsequently scored independently, according to standardized criteria [22]. Respiration was monitored throughout the night by nasal and oral thermocouples (model TCT 1R, Grass Instrument, Quincy, MA), and by nasal pressure transducer (MP45-871 ± 2 cm H_2_0, Validyne Engineering Corp.) and thoracic strain gauges. All-night recordings of hemoglobin oxygen saturation (SaO_2_) were obtained with a cardiorespiratory oximeter (Model 8800, Noonin Medical, Plymouth, MN) attached to the finger. “Apneas” were breath cessations exceeding 10 seconds; “hypopneas”, airflow reductions of ~50% with 3% reduction of SaO_2_. “Apnea-hypopnea index” (AHI) was the number of apneas and/or hypopneas/hr of sleep. “Respiratory disturbance index” (RDI) was AHI plus the number of breathing related arousals/hr of sleep – i.e., number of EEG arousals associated with reduction in airflow of ~30% without 3% reduction of SaO_2_ [23]. The RDI including nasal cannula/pressure transducer flow limitation events terminated by arousal has been previously shown to be essentially identical to the number of the esophageal manometry events terminated by arousal, which have been called Respiratory Effort-Related Arousals [24]. “Periodic leg movements” (PLMS) were counted if lasting from 0.5 to 5 seconds and in intervals of less than 90 seconds between movements. “PLMS index” was number of periodic leg movements/hour of sleep.

The next morning, subjects were evaluated by the sleep specialists of the Sleep Research and Treatment Center and were diagnosed with primary sleep disorder`(s) if they met International Classification of Sleep Disorders criteria [25]. Then, patients, who by history and/or PSG had evidence of having hypersomnias of central origin (either idiopathic hypersomnia or narcolepsy), underwent two 60 min daytime naps at approximately 9:00 AM and 12:30 PM [26]; this protocol provides a quantitative assessment of pathological diurnal sleepiness and has been suggested as an alternative to the Multiple Sleep Latency Test in the evaluation of patients with these disorders [27].

We used two PSG-based criteria to define clinically significant sleep-disordered breathing: (1) an AHI <5 events/hr with an RDI >10 or (2) an AHI ≥5 events/hr. In addition, to receive the diagnosis of SDB, patients fulfilling either criterion had to also have daytime sleepiness/fatigue and/or cardiovascular problems i.e., hypertension. Healthy controls did not fulfill criteria for any sleep diagnosis.

### Statistical analysis

Subjects were classified into four “sleep diagnosis groups” (SDB, insomnia, idiopathic hypersomnia, and narcolepsy) according to their primary diagnosis. The SDB group was comprised of patients diagnosed as having sleep disturbed breathing; the insomnia group included patients diagnosed as having insomnia as defined by ICSD-2 general criteria for the disorder; the idiopathic hypersomnia group included patients diagnosed as having idiopathic hypersomnia; and the narcolepsy group included patients diagnosed by narcolepsy with or without cataplexy. To maintain group homogeneity, we made the post hoc decision to exclude patients with more than one sleep diagnosis as follows: 3 hypersomnia patients with AHIs between 5 and 14; two narcolepsy patients – one with AHI < 5 and RDI > 10 and one with AHI between 5 and 14; six patients with insomnia (three with AHI values between 5 and 14 and three with values ≥ 15); and three patients with RLS (2 with AHIs > 15 and 1 with insomnia). Two subjects with other primary sleep diagnoses were not included in the analysis because of small cell sizes: one with sleepwalking and the other with delayed sleep-phase syndrome. Dropping these 16 subjects reduced our evaluable sample to 159.

Because sample sizes of patients with idiopathic hypersomnia and narcolepsy were small (n = 10 and 6, respectively), we did not include them in further analyses but provide data on frequency of CFS and/or FM (20% and 50% respectively; see results for breakdown of CFS and FM diagnosis) to stimulate further research on their relation to sleep disorders. We used analyses of variance to compare subjects across the remaining three study groups (healthy controls, SDB, and insomnia) as to age and BMI. We used Chi square testing to determine if rates of symptom-based illnesses varied between the sleep disorders. We used multivariate logistic regression to examine if the difference in rates of symptom-based illnesses persisted after controlling for gender, age, and RDI. The same analysis was used to assess the roles of AHI, sleep efficiency, BMI, presence of symptom-based illness and BDI score in affecting sleepiness in SDB. Analysis of covariance was used to test the differences in BDI scores between the two sleep disorders groups with or without symptom-based illnesses and healthy controls as well as if differences in BDI scores existed between patients with zero, one, two or three unexplained syndromes. A similar approach was used to determine if differences in sleepiness existed between the two sleep disorder groups with or without symptom-based illnesses. Flowcharts for the SDB and insomnia group are provided [see Tables 1 and 2]. Analysis of covariance was also used to examine differences between the two sleep disorders groups with or without symptom-based illnesses and healthy controls in PSG variables.

**Table 1:** **Detailed breakdown of characteristics of patients with SDB**

**All SDB = 122**			
**Low AHI = 36**			
Men = 18 →	No CFS/FM = 16 →	ESS ≥ 16 = 0 →	Beck < 20 = 0; Beck ≥ 20 = 0
		ESS < 16 = 16 →	Beck < 20 = 16; Beck ≥ 20 = 0
	CFS/FM = 2 →	ESS ≥ 16 = 1 →	Beck < 20 = 1; Beck ≥ 20 = 0
		ESS < 16 = 1 →	Beck < 20 = 1; Beck ≥ 20 = 0
Women = 18 →	No CFS/FM = 14 →	ESS ≥ 16 = 1 →	Beck < 20 = 1; Beck ≥ 20 = 0
		→	Beck < 14 = 0; Beck ≥ 14 = 1
		ESS < 16 = 13 →	Beck < 20 = 12; Beck ≥ 20 = 1
		→	Beck < 14 = 10; Beck ≥ 14 = 3
	CFS/FM = 4 →	ESS ≥ 16 = 3 →	Beck < 20 = 2; Beck ≥ 20 = 1
		ESS < 16 = 1 →	Beck < 20 = 1; Beck ≥ 20 = 0
**High AHI = 86**			
Men = 65 →	No CFS/FM = 59 →	ESS ≥ 16 = 7 →	Beck < 20 = 6; Beck ≥ 20 = 1
		→	Beck < 14 = 5; Beck ≥ 14 = 2
		ESS < 16 = 52 →	Beck < 20 = 49; Beck ≥ 20 = 3
	CFS/FM = 6 →	ESS ≥ 16 = 1 →	Beck < 20 = 1; Beck ≥ 20 = 0
		→	Beck < 14 = 0; Beck ≥ 14 = 1
		ESS < 16 = 5 →	Beck < 20 = 4; Beck ≥ 20 = 1
		→	Beck < 14 = 3; Beck ≥ 14 = 2
Women = 21 →	No CFS/FM = 17 →	ESS ≥ 16 = 5 →	Beck < 20 = 5; Beck ≥ 20 = 0
		→	Beck < 14 = 4; Beck ≥ 14 = 1
		ESS < 16 = 12 →	Beck < 20 = 12; Beck ≥ 20 = 0
		→	Beck < 14 = 10; Beck ≥ 14 = 2
	CFS/FM = 4 →	ESS ≥ 16 = 2 →	Beck < 20 = 1; Beck ≥ 20 = 1
		→	Beck < 14 = 0; Beck ≥ 14 = 2
		ESS < 16 = 2 →	Beck < 20 = 2; Beck ≥ 20 = 0

We are presenting data stratifying the ESS data by scores 16 or higher; this was the median value for SDB patients fulfilling diagnostic criteria for CFS and/or FM. We are presenting Beck data two ways: with the cut off at 20 [moderate depressed mood] and at 14 [mild depressed mood] if data differed from those using the 20 cut off.

**Table 2:** **Detailed breakdown of characteristics of patients with insomnia**

**Insomnia (n = 21)**			
**Men (n = 8)** →	No CFS/FM =4 →	Beck ≥ 20 = 1 →	ESS ≥ 16 = 0; ESS < 16 = 1
		Beck < 20 = 3 →	ESS ≥ 16 = 0; ESS < 16 = 3
	CFS/FM = 4 →	Beck ≥ 20 = 2 →	ESS ≥ 16 = 1; ESS < 16 = 1
		Beck < 20 = 2 →	ESS ≥ 16 = 0; ESS < 16 = 2
**Women (n = 13)** →	No CFS/FM =7 →	Beck ≥ 20 = 1 →	ESS ≥ 16 = 0; ESS < 16 = 1
		Beck < 20 = 6 →	ESS ≥ 16 = 0; ESS < 16 = 6
	CFS/FM = 6 →	Beck ≥ 20 = 3 →	ESS ≥ 16 = 1; ESS < 16 = 2
		Beck < 20 = 3 →	ESS ≥ 16 = 1; ESS < 16 = 2

We are presenting the Beck data stratified by scores of 20 or higher. An analysis stratifying with scores of 14 or higher produced very similar results. We are presenting data stratifying the ESS data by scores 16 or higher; this was the median value for SDB patients fulfilling diagnostic criteria for CFS and/or FM.

Data in the text are presented as mean (SD). P < 0.05 was considered statistically significant.

## Results

### Sleep disorders

Of the 159 evaluable patients with sleep complaints who underwent PSG, 122 (77%) patients were diagnosed with sleep disturbed breathing, 21 (13%) with insomnia (for SDB and insomnia, see Table 3), 10 (6%) with idiopathic hypersomnia, and 6 (4%) with narcolepsy.

**Table 3:** **Demographics and rates of fibromyalgia and chronic fatigue syndrome occurring alone or together**

	**Healthy controls (n = 39)**	**SDB (AHI 5–14; n = 36)**	**SDB (AHI >14; n = 86)**	**SDB (n=122)**	**Insomnia (n=21)**	**SDB vs. Insomnia (p value)**
Men, % (n)	44 (17)	50 (18)	76 (65)	68 (83)	38 (9)	.009
Age (SD), y	42.9 (15.0)	50.3 (11.2)	51.8 (12.0)	51.4 (11.7)	37.8 (8.7)	.000
BMI (SD), kg/m^2^	25.1 (4.0)	30.7 (5.0)	31.6 (4.2)	31.3 (4.5)	29.4 (5.2)	.070
AHI (SE)	1.8 (0.3)	9.6 (0.6)	39.7 (2.5)	30.8 (2.1)	1.9 (0.3)	.000
RDI (SE)	2.8 (0.4)	12.2 (0.8)	42.2 (2.4)	33.3 (2.1)	2.7 (0.4)	.000
FM only, % (n)	0 (0)	0 (0)	0 (0)	0 (0)	14 (3)	.003
CFS only, % (n)	0 (0)	14 (5)	8 (7)	10 (12)^1^	19 (4)^3^	.189
CFS + FM, % (n)	0 (0)	3 (1)	4 (3)	3 (4)^2^	14 (3)^4^	.065
No CFS or FM, % (n)	100 (39)	83 (30)	88 (76)	87 (106)	52 (11)	.001
With CFS or FM, % (n)	0 (0)	17 (6)	12 (10)	13 (16)	48 (10)	.001

SDB = sleep disordered breathing; BMI = body mass index; FM = fibromyalgia; CFS = chronic fatigue syndrome.

^1^One man in this group had well controlled diabetes and well controlled hypertension.

^2^One woman in this group had well controlled diabetes and well controlled hypothyroidism.

^3^One man in this group had well controlled hypothyroidism.

^4^Two women in this group had well controlled hypertension.

Of the patients with SDB, 36 (30%) had AHIs ranging from 5 to 14 while 86 (70%) had AHIs equal or greater than 15; none had AHI < 5 with RDI > 10; therefore, all fulfilled criteria for obstructive sleep apnea. The only difference between the two SDB groups was in gender (see Table 3): 50% of the 36 SDB patients in the group with lower AHIs were women compared to 24% of the 86 SDB patients in the severe group (Fishers test = .001). Comparing the combined SDB patient group with that of patients with insomnia, those with SDB were significantly older and more likely to be male (Table 3).

### Symptom-based syndromes – fibromyalgia, chronic fatigue syndrome and irritable bowel syndrome

As defined by our intake criteria, none of the controls had CFS or fibromyalgia. In contrast, patients with SDB and insomnia had appreciable rates of CFS and/or FM – 13% and 48%, respectively (see Table 3). However, while insomnia patients showed a relatively even split between CFS and FM, SDB patients showed predominately CFS with low rates of FM – similar to the 3% rate seen in the general population [28]; in contrast to the insomnia group which had patients with FM only, the few cases of FM that did occur in the SDB group occurred concomitantly with CFS. The apparent difference in rates between SDB and insomnia remained significant after controlling for gender and age (p = 0.012). There was no substantial difference in constitution of symptom-based diagnoses between patients with lower AHIs compared to those with high AHIs (Low AHI: 0% FM, 14% CFS, 3% CFS + FM; High AHI: 0% FM, 8% CFS, 4% CFS + FM).

Using data from the entire sample of 159 evaluable patients, the diagnosis of CFS alone occurred most often (n = 18), CFS and FM next most often (n = 8), and FM alone least often (n = 5); this also is different from what one might expect in that rates of FM in the general population are ten times higher than those for CFS. Of the 10 patients with idiopathic hypersomnia, none had CFS only or CFS + FM, and 2 had FM only. Of the 6 patients with narcolepsy, 2 had CFS only, 1 had CFS + FM and none had FM only.

One control subject had IBS alone. Nine of the SDB patients had IBS [7 alone, 1 with CFS, and 1 with CFS + FM]; three of the insomnia patients had IBS [1 alone; 1 with FM and, 1 with CFS + FM].

### Mood

Mood as measured by BDI did not differ significantly for sleep diagnosis groups without symptom-based syndromes compared to healthy controls (2.6 ± 3.0 [SD], healthy controls; 6.2 ± 6.9, SDB; 8.9 ± 7.8, insomnia). However, when SDB or insomnia co-existed with CFS and/or fibromyalgia, BDI scores were significantly higher (14.0 ± 11.0 and 20.9 ± 11.6, respectively) than in controls (2.6 ± 3.0; all p’s = 0.000) indicating more depressed mood; this effect remained after controlling for gender and age.

As the number of symptom-based diagnoses increased, BDI scores increased monotonically: lowest in patients with no symptom-based diagnoses (5.5 ± 5.8 [SD]), significantly higher in patients with one (11.9 ± 11.7; p =0.000), significantly higher yet in patients with two (19.0 ± 10.2; p < 0.05) and higher again, albeit not significantly, in patients with three symptom-based illnesses (30.5 ± 3.5).

### Subjective daytime sleepiness

As expected, patients with insomnia or with SDB were sleepier on the Epworth Sleepiness Scale than controls, respectively (9.4 ± 5.4 [SD]; 9.9 ± 5.2, 6.1 ± 3.8; p = 0.044 and p = 0.000, respectively). The existence of co-morbid symptom-based syndromes affected sleepiness scores for patients with SDB but not for those with insomnia: SDB patients with CFS or CFS + FM had significantly higher ESS scores than those without (13.3 ± 4.5 and 9.4 ± 5.1, respectively, p < 0.05); of the 16 patients in this group, 63% had ESS scores >10 (Mdn = 16; IQR = 14.8, 17.3). The significant difference between SDB patients with and without symptom-based syndromes disappeared when Beck Depression Scores and sleep efficiency values were entered into the regression – leaving these latter variables significant (p = 0.003 and p = 0.003, respectively).

### Sleep characteristics

The existence of CFS and/or FM did not alter standard sleep characteristics of sleep duration and architecture, and only minor differences in sleep characteristics were seen (data not shown).

## Discussion

The results of this study lead to two main discussion points – whether CFS and FM are the same or different illnesses and whether patients with these clinical diagnoses may actually have SDB as the cause of their symptoms.

Concerning the first point, a review of the literature suggested that FM and CFS might be different disease processes in that SDB was reported to be common in FM while several studies, including our own [9-11], indicate SDB is not common in CFS. One study reported finding SDB to occur in men but not women with FM [13], and two others reported finding SDB at high rates in women with FM [14,15]. We did not find an increased rate of FM in our earlier study of sleep characteristics in patients with CFS alone or CFS plus FM [11]. Two small studies used the approach we had taken here of evaluating patients with PSG-proven SDB for FM. One study found no increase in FM in SDB [12] while the second did [16]. We expect these discrepancies may be due to methodological differences related to our excluding patients with multiple sleep diagnoses and/or issues related to sample size. In contrast to those reports reporting an increased rate of FM in SDB, we found an actual dissociation between the diagnosis of FM and SDB. None of our SDB patients had FM alone and only 3% – the same rate as seen in the general population for FM [28] – had FM with CFS.

Because CFS and FM co-exist for a substantial number of patients, some researchers have suggested both ailments fall within the category of somatic amplification, having functional or psychogenic rather than organic bases [3,4]. The data reported here provide two pieces of evidence against that interpretation. First, if CFS, FM and IBS were all variants of a similar psychological process, Beck scores should be the same for patients bearing the diagnosis of one, two or three of these symptom-based syndromes. This was not the case: Beck scores increased monotonically with frequency of symptom-based syndromes – a finding we have reported earlier [29].

Second, if CFS and FM were essentially the same illness, then rates of both illnesses should co-vary and rates of each should not differ between different primary sleep disorders. This was not the case. Rates of CFS and FM did covary in insomnia, but rates of CFS and FM diverged in SDB with these patients having high rates of CFS but rates of FM at the population norm. Finding this difference suggests that CFS and FM have different pathophysiological underpinnings – at least in the face of SDB. Therefore these data argue against the “unitary” hypothesis that CFS and FM are variants of a functional somatic syndrome due to a psychologically based amplification of symptoms that every person experiences. That conclusion must be tempered by the possibility that such a mechanism could produce different symptoms in different patients.

Despite the small sample size, finding that narcolepsy patients had predominantly CFS and not FM further supports the idea that CFS and FM are not necessarily the same. The fact that differences do exist between CFS and FM supports the continued use of individual case definitions for each disorder and argues against modifying diagnostic criteria for FM [30] to bring the two disorders closer together.

The second discussion point has to do with the issue of ruling out underlying SDB as a possible, but unappreciated, cause of apparent chronic fatigue syndrome. Current guidelines do not recommend a formal sleep evaluation including PSG as part of the work up to rule out medical causes of fatigue before making the diagnosis of CFS. However, we found that 13% of patients with PSG-proven SDB fulfilled case criteria for CFS. In view of the fact that the community prevalence of CFS is 0.42% [31], the rate of 13% is greatly increased and of clinical importance. This finding leads to two research questions. First, is it possible to predict the existence of SDB in patients receiving the diagnosis of CFS? Being able to make this prediction would lead to PSG to corroborate the existence of this sleep disorder and then to try to treat it.

Excessive daytime sleepiness as assessed by the Epworth Sleepiness Scale has turned out to be a disappointing predictor of SDB in that the majority of SDB patients have Epworth scores in the normal range [32]. However, the predictability of elevated Epworth scores in identifying SDB is greatly improved for those SDB patients who also fulfill criteria for CFS. More than half of the 16 patients in this group had Epworth scores >10 with the mean, median and 75th percentile being 13.3, 16 and 17.3, respectively. In considering these data, it would be important to know Epworth data for CFS patients without SDB. While our study did not provide these data, two studies do provide such data for samples of 24 and 415 CFS patients, respectively (mean = 9.4; SD = 4.5 [33]; mean = 10.5; SD = 5.5 [34]). Since available data indicate SDB to be at population rates for CFS samples, we accept the result of these studies as being typical of CFS patients without SDB. Epworth scores were significantly lower by t-tests (p < 0.05) than in the sample with SDB reported here. Thus, patients with SDB who also fulfill criteria for CFS alone or CFS with FM have more daytime sleepiness than CFS patients without SDB.

The second research question is whether treating the SDB in CFS-SDB patients with CPAP would produce symptomatic improvement. While the original case definition for CFS did not require PSG in the work up of patients with severe and chronic fatigue, the 2003 discussion of shortcomings in that case definition [35] emphasized that “assessment of sleep must detect treatable primary sleep disorders and evaluate sleep-related symptoms that may be part of CFS”. Our finding that 13% of patients with SDB also fulfilled criteria for CFS supports that conclusion. The data presented here suggest an algorithm to help the physician determine which CFS patient should undergo PSG based on his/her Epworth Sleepiness Score. We recommend Epworth scoring on all patients being evaluated, and then, to reduce the risk of a negative sleep study, PSG should be considered for the patient who fulfills criteria for CFS and who has an Epworth score of 16 or above – i.e., at and above the 50th percentile for this group; based on Epworth data from 415 CFS patients [34], approximately 13% of all patients would fall into this category. Using these criteria to perform PSG in patients with the diagnosis of CFS will allow the clinician to make the diagnosis of SDB and then determine whether treating it may greatly reduce the complaint of severe fatigue. This important study remains to be done.

The use of antidepressants as a second possible treatment for this group of patients emerges from our finding an association of Epworth scores in patients with SDB and CFS with high BDI scores, a measure of depressed mood. The link between depression and excessive daytime sleepiness has been shown in general population samples as well as in those comprised of SDB patients [36-38]. This treatment may be most effective in the subgroup of patients with both high Epworth and high BDI scores.

Strengths and Weaknesses: A major strength of this study was the fact that we had a large sample size of 122 patients with SDB. Having so many SDB patients provided another strength in that we had an adequate sample to examine whether patients with a milder form of SDB might differ from those with more severe SDB in rates of CFS and/or FM. They did not. Being able to look across the breadth of SDB helped rectify another weakness – that FM is predominantly a disease of women and thus might not be seen in adequate numbers in an SDB population; however, the data were essentially the same for the two SDB groups even though the gender prevalence in the two groups did differ. One potential weakness had to do with our sample – people with sleep complaints referred for evaluation in a sleep lab. While the complaint of unrefreshing sleep is among the most common symptoms of CFS [39], it is not endorsed by all patients and so the patients studied here may have been biased toward those with identifiable sleep disorders; conversely, patients with CFS may have unrefreshing sleep stemming from their other symptoms/problems such pain, sore throat, headaches. Sample size was also a weakness – at least for the small groups with IH and narcolepsy. Nonetheless, finding high rates of CFS in narcolepsy was not unexpected as these patients are notable for having marked daytime sleepiness, but the result does suggest the possibility of misdiagnosis for some patients thought to have CFS – further support for doing PSGs in selected cases of excessively sleepy patients with severe fatigue. A next step for researchers interested in the relation between sleep and symptom-based illness will be to expand the sample size of patients with these two sleep diagnoses.

## Conclusions

A substantial number of patients with sleep disturbed breathing fulfill criteria for the diagnosis of chronic fatigue syndrome, but not for fibromyalgia. In contrast, patients with insomnia fulfill criteria for both chronic fatigue syndrome and fibromyalgia. The fact that rates of CFS and FM differ in sleep disturbed breathing suggests differences in the underlying pathophysiology between the two illnesses. It will be important to identify those CFS patients who may have sleep disturbed breathing. To do this, the authors suggest that CFS patients complete Epworth Sleepiness Scales. Those with values of 16 or greater should undergo formal sleep evaluation to determine if they have the potentially treatable condition of sleep disturbed breathing.
